# Coping Work Strategies and Job Satisfaction Among Iranian Nurses

**DOI:** 10.5812/ircmj.17779

**Published:** 2014-06-05

**Authors:** Shahrzad Ghiyasvandian, Addis Adera Gebra

**Affiliations:** 1 Medical Surgical Department, Faculty of Nursing and Midwifery, Tehran University of Medical Sciences, Tehran, IR Iran

**Keywords:** Job Satisfaction, Nursing, Iran, Nurses

## Abstract

**Context:**

Nursing is a stressful job that could create physical and psychological disorders. Many studies presented information on stress, effects of coping strategies, and job satisfaction of nurses within health setting. We aimed to identify and describe nursing stresses, coping strategies and job satisfaction of Iranian nurses who are working or worked in different wards.

**Evidence Acquisition:**

In this review, we studied peer-reviewed journal articles on the field of stress, coping strategies and job satisfaction in nursing practice, especially Iranian nurses, which were published between 2000 and 2013. In this regard, we searched databases of PubMed, Elsevier, Google, BMJ, PMC, and MEDLINE.

**Results:**

The majority of the studies (60%) had analyzed the effect of coping strategies, experiences and perception of job-related stresses in Iranian nurses working in hospitals. In some of the reviewed studies (60%), the majority of the samples enrolled Iranian nurses. Forty percent of studies selected a maximum sample size of 565 (44%) participants in 2011. Nursing stress scale employed at 30% of the studies was the most commonly used strategy. This reviewed studies also revealed a combined measurement (60% of studies), based on categorical stress measurement, effects of coping strategies, and job satisfaction methods. Three studies explored the relationship between job stress and job satisfaction. For instance, the majority (74.4%) of nurses reported job satisfaction.

**Conclusions:**

Effect of coping strategies and job satisfaction on Iranian nurses is a well-accepted issue and has important positive outcomes on several areas of health discipline.

## 1. Context

Stress is not inherently harmful; however, everyone’s cognitive appraisal, perceptions, and interpretations give meaning to events and determines them as negative or positive. Personality traits also influence the stress equation as what may seem overtaxing to one person is exhilarating to another (1).

Although stress is typically considered a negative feeling, it could be neutral or positive depending on the individual and the specific situation. Eustress is defined as the positive stress that allows an individual to remain productive regardless of changes in the external environment.

As nurses cope with staffing shortage, rapid changes in patients’ conditions and technological advances, higher stress levels may appear in their work environment. This can interfere with their productivity and negatively affect the level of care provided to the patients (2). According to the American Holistic Nurses Association, the major factors that contribute to stress include inadequate staffing or high workload, the nurses’ relationships with other medical staff, leadership style and support, and coping with the emotional needs of patients (3).

In addition, nurses are one of the professional groups most vulnerable to occupational stress, as They often encounter stressful situations due to the special demands of their profession (4). Nurses’ work stressors might contribute to their decreased job satisfaction, which might also lead to higher turnover rate and lower nursing care quality for patients (5). Nurses’ stress is defined as “The emotional and physical reactions resulting from the interactions between the nurse and her/his work environment where the demands of the job exceed capabilities and resources” (6).

Many studies have focused on work stress among nurses because they work in a stressful environment, which has detrimental effects on their mental and physical health, productivity and efficiency at work, absenteeism, as well as outcomes for patients such as increased mortality and patient’s dissatisfaction (7).

Stress levels are directly related to job satisfaction (9). Some research results have indicated that 21.67% of nurses showed psychological symptoms (10). In spite of regulations at work, many events and risk factors are unavoidable; special skills are important to improve daily activities in clinical settings (11). Nurses who have received strong support from their coworkers and supervisors experienced lower levels of job stress and emotional exhaustion (8).

Recent studies have revealed the importance of social support in coping with job stress and preventing emotional exhaustion. Social support reduces both the intensity and consequences of stressors (8). More recently, the problem of nursing shortage and its consequences have worsened the situation (12). Levels of occupational stress among nurses in hospitals are reported to be significantly higher than nurses in other health care environments (13). Studies conducted on nurses of South Spain hospitals showed that social support had a significant protection effect on the level of stress and emotional exhaustion experienced by the nurses at work.

Some factors that affect occupational stress include working conditions, relationships at work, role conflict and ambiguity, organizations at work, working climate, work–home interface, career development, and the nature of the job (14, 15). In this regard, stress management interventions have been proposed and their effectiveness in reducing stress and improving physical and mental health among medical staff has been investigated (16, 17).

In a study of job stress among hospital nurses, 27% of the participants experienced stress and 35% reported consulting a doctor in the past 6 months. Also, nurses experienced job stresses differently (18). To minimize stress, people use coping strategies, which are defined as cognitive and behavioral efforts to dominate, tolerate, or reduce demands (19). For example, in a study on the coping strategies employed by preoperative nurses, 25% of females demonstrated avoidance strategies, while 83% of males used problem-solving strategies (20).

How people use coping strategies are, in part, determined by their internal and external resources, which include health, beliefs, responsibilities, support, social skills, and material resources (19). The findings of this review provide better understanding of the major causes of job stress and its effects on nurses in health institutes and their services. In other words, the reliable information gathered in this review has provided helpful insight to different health settings in Iran when dealing with job stresses among nurses.

In this paper, we reviewed all the studies that quantitatively analyzed nursing stress, effect of coping strategies and job satisfaction among Iranian nurses working or training in hospitals. In addition, we chose only those studies that used standardized instruments for assessing the stressful situations, effect of coping strategies and their job satisfaction. This review considers fewer studies compared with the similar studies.

This paper aims to identify and describe nursing stress, effect of coping strategies and job satisfaction of Iranian nurses who are working in hospitals or having work experience in different wards.

## 2. Evidence Acquisition

We conducted a systematic review of the research literature in the field of stress, coping strategies and job satisfaction in nursing practice. In this regard, we investigated the PubMed, Elsevier, Google, BMJ, PMC, MEDLINE, and Psych info search engines. This method was used to determine the accuracy of the results through comparing them in the related articles from various sources. This review is based on peer-reviewed journal articles on “nursing stress”, “effects of coping strategies”, and “job satisfaction in Iranian Nurses” that were published between 2000 and 2013. This time span was chosen because up-to-date information is important to justify and address the objective of the review articles.

After assessing all titles and contents of the articles, the relevance of the articles to address the objectives of this study were considered and evaluated. The terms used for searching related articles were “stress”, “coping strategies”, “job satisfaction”, and “work environment”. Also “nurses” and “Iran” were used together to search the articles from different websites. In the first step, a total of 26 articles were found. The titles, summary or abstract, and the whole body of each article were then checked for their relevance and suitability to be included in the analyses. Finally, a total of 10 articles were found to be eligible (Table 1).

First, we used the Effective Public Health Practice Project Quality Assessment Tool (EPHPP) (21) to assess the quality of quantitative research in this review. This instrument had ordinal scoring for the following components: selection bias, study design, confounders, blinding, data collection methods, withdrawals and dropouts. Each component was rated strong, moderate, or weak, and these ratings were also combined into a global quality rating (strong, moderate or weak). Two reviewers assessed the quality, and discrepancies were resolved by discussion.

**Table 1.** Studies Evaluating the Nursing Stress: The Effects of Coping Strategies and Job Satisfaction in Iranian Nurses^a^

Study, Setting, Ward	Sample Size	Method Study Design	Technique	Inclusion	Analysis	Level of Significance
**Gholamzadeh et al. 2011, 3 large teaching hospitals, Iran, Emergency (22)**	90	Descriptive survey	Volunteered to enrolled in the study	All the responders of the study held a bachelor’s degree	Descriptive statistics	Data were cross tabulated and chi-square test of significance was calculated
**Moneini et al. 2011, 2 Hospitals, Hamandan Northwest, Iran, two different hospital of different wards (23)**	58 (all F)	Quasi experimental	Randomly selected as an Intervention (Fatemieh) and control hospital (Shahid Beheshti)	All nurses participated voluntarily in the study	t-test, Mann-Whitney and Wilcoxon statistical test	95% significant level
**Golshiri et al. 2012, 2 Hospitals Emergency ward; Female Clerk Hospital (31)**	84	Cross-sectional	Census method	Willingness to participate in the study. Having the minimum saliva flow (0.1 mL/min). All female nurses in the three working shifts of the hospital emergency ward	Independent t test, variance analysis , Partial correlation	For all test P = 0.05
**Shakerinia et al. 2010, Hospital, Iran, Emergency; Surgical; Gynecology; pediatrics; and cardiology (24)**	124	Correlational descriptive/comparison	NA	Experienced nurses who have been working in different wards	Correlation, (Analysis Of variance (ANOVA), Tukey, Regression	P = 0.05
**Dargahi et al. 2011, 15, Teaching hospitals Tehran, Iran, All Wards (25)**	389	Cross sectional descriptive and analytical	Randomly stratified sampling	Nursing personnel with bachelor and master degrees four regions of Tehran and frequency of the hospital in each region.	t-test, ANOVA, statistical method	NA
**Mojdeh et al. 2007, 1-Alzahar Hospital, Different Wards of Alzhara hospital (29)**	216	Cross-sectional	Random convenience sampling	Working in different wards of Al-Zahra hospital with associate’s bachelor’s and master’s degree	Kruskal, Wallis , Mann-Whiney tests	P < 0.05
**Jafari et al. 2012, University hospital of Zanjan, all wards (26)**	241	Analytical descriptive cross-sectional	Proportional to size stratified method	Agreed to voluntarily participate, individuals experiencer loss of family member, divorce, severe illness in the last 6 months not included	Enter method regression, Spearman’s Kuruskal, Wallis and, 1-way ANOVA	All data are expressed as Mean ± Standard Deviation
**Jannati et al. 2011, Hospital (various Role and setting), all hospital wards (27)**	28	Qualitative study exploration	Willingness of participants	Willingness to participate, experience of working as a nurse for more than one year	Content analysis, method developed by “Strauss and Cobin (1998)”	Check with some of the observers clinical nurses who conformed the fitness of the results as well with axial coding at the end of the selected coding phase, the core variables was identified
**Mehrabi et al. 2012, Al-zaha Hospital, Intensive Care Unit (ICU) (28)**	34 (all F)	Quasi-experimental (Two-group, single stage, before and after quasi experimental study)	Hospital were selected as subject, with Inclusion criteria	Having at least bachelor in nursing, history of joining similar researches. Having 1 year work experiences in ICU at the time of study	Descriptive and inferential (t test) statistical tests	
**Adib-Hajbaghery et al. 2012, Kashan university of Medical science hospital,** **Ward not specified (30)**	19	Qualitative	One or more occasions with respect to environmental factors, patience, and the willingness of the participants	Participant had at least 2 years of experience, No self-reported history of mental illness	Content analysis according to Krippendorff method	

^a^Abbreviations: NA, not applicable; ANOVA, analysis of variance; F, female

### 2.1. Inclusion and Exclusion Criteria

The authors read the titles and decided to include articles in the review regarding the following criteria:

1. the study included one or more instruments to gather information on stress, effect of coping strategies and job satisfaction in Iranian nurses,
2. inclusion of only nursing professionals who had work experience and were working in health Institutes in Iran.

Articles published between 2000 and 2013, and those published in English were eligible for the analysis.

Studies that did not meet these criteria were excluded. This review is focused on the comparison of levels and sources of stress, effects of coping strategies and their job satisfaction on Iranian nurses in different hospitals. Furthermore, the review identified the main characteristics of Iranian nurses’ stress, effects of coping strategies and job satisfaction.

However, all abstracts were excluded from this review article. For instance, articles about instrument development, stress in specific populations, children, adolescences, pregnant women, parents care givers, occupations other than health care, articles about nursing students, and countries other than Iran, were excluded from this review.

## 3. Results

Out of 10 reviewed studies, 6 (60%) analyzed the effect of coping strategies and experiences and perception of job-related stresses in Iranian nurses who were working in hospitals (13, 22-26). Two studies (20%) analyzed the effect of coping strategies and yoga employed among Iranian nurses in hospital wards (27, 28). The remaining studies (20%) analyzed the effect of a cognitive–behavioral stress management training program (23), and assessed the relationship of nurses’ stresses to environmental and occupational factors (29).

Three studies (30%) included samples of nurses who had more than 1-year’s work experience, at least 2 years working in different wards (24, 27, 28), and voluntarily participated in the studies (22, 27, 30). Two (20%) studies, enrolled nurses working in different wards and held an associate’s, bachelor’s, or master’s degree (25, 29). Two studies (20%), however, enrolled nurses working in different wards and held a bachelor’s degree (22, 28).

The selection of participants was made using both random sampling and non-random sampling methods such as volunteering, convenient and purposive sampling method. The majority of studies reported techniques of participant selection; the largest percentage of studies (40%) relied on volunteerism (22, 27, 30). Five out of 10 studies used equal random selection (23), census (31), random stratified sampling (24), proportional sampling (26), and selective hospitals (28). However, the sampling technique was not clarified in one study (10%).

Eight of 10 reviewed studies (80%) utilized quantitative approaches to address the research questions (22-26, 28, 29, 31). Two studies (20%) used only qualitative approach to address the same questions (27, 30). In the reviewed studies, the majority of the samples (60%) involved Iranian nurses enrolled in work experience (23, 25-27, 29-31). Four studies (40%), in the remaining cases, (22, 24, 28, 31) compared Iranian nurses who were working in emergency only (1) both emergency and clerk staff (31), emergency, surgical, gynecological and cardiology wards (24), and intensive care units (ICU) (28).

 The majority of the reviewed articles (40%) selected a maximum sample size of 565 (44%) participants in 2011 (22, 23, 25, 27). The next 40% selected a sample size of 378 (29.46%) participants in 2012 (26, 28, 30, 31). The remaining studies (20%) had a sample size of 216 (16.85%) in 2007 (29), and 124 (9.70%) participants in 2010 (24) respectively. Moreover, some non-medical and medical staff such as administrators, and doctors were not included in the reviewed studies.

As shown in Table 2, this study sought to generalize results from the mentioned articles by identifying a wide variety of coping strategies and job satisfaction that might be used by nurses. These results show the different instruments used to measure Iranian nurses’ stresses and their work environment. A nursing stress scale, which accounted for 30% of articles on stress measurement was the most commonly used strategy (23, 25, 29), followed by various measurements in each of the remaining studies such as Strauss and Corbin’s grounded theory (10%) (27), coping stress revised questionnaire (10%) (28), Lazarus standard questionnaire (10%) (22), Persian short version of Generic Job Stress National Institute for Occupation Safety and Health (NIOSH) (10%) (31), Job stress (10%) (24), Toft Gray and Anderson’s tool (10%) (26), and open Semi-structured Interview (10%) (30).

**Table 2.** Nursing Stress, Coping Strategies and Job Satisfaction Instruments, Purpose of Instruments, and Their Results Used in Iranian Nurses Stress, Coping Strategies and Job Satisfaction^a^

Study, Reference	Aim of Study	Instruments/Measurement	Purpose of Instruments	Study Finding/Result
**Gholamzadeh et al. 2011 (22)**	To investigate the sources of job stress and the adopted coping strategies of nurses who were working in the emergency department	Self-administrated questionnaire, nurses profile, Lazarus standard questionnaire	To identify the source of job stress, nurses profile, to determine the type of coping strategies	Relationship between the use of the confrontive coping scale and sex of the participants, relationship in the use of any coping scale and other demographic variables considered, the majority (74.4%) of nurses reported satisfaction
**Moeini et al. 2011 (23)**	To determine the effect of a cognitive-behavioral stress management training program based on PRECEDE Model on stress reduction among nurses.	Demographic, nursing stress scale, an author developed questionnaire based on PRECEDE model/components	To evaluate the present status	A significant difference was found in PERCED model constructs and stress management behaviors in intervention group compared to control group after training intervention (P < 0.001), the baseline score average of job stress was 113.0 and 109.8 for intervention and control group (P = 0.250), after intervention, core average of job stress decrease to 94.0 inexperience mental group while that of control group respectively unchanged (109.2), (P < 0.001)
**Golshiri et al. 2012 (31)**	To evaluate and compare the job stress of female nurse working in emergency wards and female clerks, to analyze the possible relationship between the stress level and level to saliva secretary IgA (SIgA)	Persian short version of generic job stress questionnaire of the National Institute for Occupational Safety and Health (NIOSH), Enzyme-linked Immunosorbent Assay (ELISA)	To measure stress level, to determined (Secretory Immunoglobulin A (SIgA level)	The results showed a negative correlation between the levels of saliva SIgA and job stress (P = 0.02 and r = 0.203), the level of saliva SIgA in nurse and clerks were 338.04 ± 380.03 and 706 ± 354.70 respectively (P < 0.001), the mean score of job stress was 97.30 and 91.85 in nurses and clerks, respectively (P = 0.016)
**Shakerinia et al. 2010 (24)**	To examine relationship between job stress and resiliency with burnout among nurses in Rasht state hospitals	Demographic, job stress, resiliency, Burnout questionnaires	Stress assessment, Burnout identification	A significant positive correlation between job stresses and nurses, a significant difference among the nurses who work at emergency and Gynecology wards, there was not a significant difference among nurses at cardiology and pediatrics
**Dargahi et al. 2011 (25)**	To measure the life change units as stressors among Iranian hospital nurses by LCU	Stress scale	Their life change units	Positive correlation between the nurses LCU rating and gender (P = 0.013)and their degrees (P = 0.021), the female nurses and the nurses with BSc degree received of hazards of work place as statistically significantly more stressful than male and M.Sc. nurses
**Mojdeh et al. 2007 (29)**	To assess relationship of Nurses’ stress with environmental and occupational factors	Demographic information, identifying stress levels/nursing stress scale	To identify degree of stress level	There was significant correlation with stress level, and job satisfaction and leisure, there was not significant correlation with stress level and some demographic information.
**Jafari et al. 2012 (26)**	To determine the quality of life of nurses and whether there is any relationship between occupational stress and quality of life	Toft Gray and Anderson’s tool	To identify stress and degree of quality of life	There was no significant correlation between quality of life or occupational stress and factors like position, shift, ward, experiences, time off, over time hours, interest in desertion and education
**Jannati et al. 2011 (27)**	To explore the coping strategies employed by the Iranian clinical nurses in-Depth	Using strolls and Corbin’s grounded theory approach focusing on the process of coping with stress, how Iranian clinical nurses cope with Job stress	To measure coping mechanism, to identify stress and related with Job	The core category was “on the route of coping “which contained six categories compromising work management, self-control, emotional, spiritual, cognitive, and Interactional strategies.
**Mehrabi et al. 2012 (28)**	To investigate the effect of yoga on stress coping strategies among nurses working in Intensive Care Units (ICUs)	Demographic characteristics, coping stress revise questionnaire	To measure coping skills, to investigate the method of response to stress.	A significant difference in the mean scores of coping strategies of stress focus, emotion focus, and ineffectiveness after yoga exercise compared with those before the yoga sessions, the highest application of stress coping strategies was for inconsistent stress coping strategy.
**Adib-Hajbaghery et al. 2012 (30)**	To understand the nurses’ experiences and perceptions of job, related stress	Open semi-structured interviews	To measure degree of psychological feeling or experiences. Job related stress.	The finding was based on categories: being in consistently alarm situation, lack of experience, dignity and social status, shortage of nurses, irregularities in the organization, directors of nursing performance, unreliable relations among colleagues, and the patients conditions all have effects on the nurses level of professional stress

^a^ Abbreviation: PERCED, educational diagnosis and evaluation model

The reviewed studies also showed a combined measurement based on categorical stress measurement, effects of coping strategies and job satisfaction methods, which accounted for 60% of studies (26, 28, 30, 31), and was the most commonly used measurement strategy, followed by single categories of measurement of stress 40% (25-27, 30).

 Three of the studies explored the relationship between job stress and job satisfaction. For instance, in the study by Goholamzadeh et al. (2011) the majority (74.4%) of nurses reported that they were satisfied with their job (22). The highest application of stress coping strategy was for inconsistent stress coping strategy (28). The baseline score average of job stress was 113.0 and 109.8 for intervention and control groups, respectively (P = 0.250). After the intervention, the average score of job stress decreased to 94.0 in the experimental group, while in the control group, scores were unchanged (109.2) (P < 0.001) (23).

 The results showed a negative correlation between the levels of saliva Immunoglobulin A (SIgA) and job stress (P = 0.02 and r = 0.203). The mean level of saliva Immunoglobulin A (SIgA) in nurses and clerks were 338.04 ± 380.03 and 706 ± 354.70 respectively (P < 0.001). The mean score of job stress was 97.30 and 91.85 in nurses and clerks, respectively (P = 0.016) (31).

There was no significant correlation between stress level and some demographic information (26). Similarly, there was no significant correlation between quality of life or occupational stress and factors like position, shift, ward, experiences, time off, overtime hours, interest in desertion and education (27).

Adib-Hajbaghery (2012) examined the core category of “ways of coping”, which contained six categories of compromising work management, self-control, emotional, spiritual, cognitive, and interactional strategies (27, 30). The findings were based on these categories: “being consistently in an alarming situation”, “lack of experience”, “dignity and social status”, “shortage of nurses”, “irregularities in the organization”, “directors of nursing performance”, “unreliable relations among colleagues”, and “the patients’ conditions all have effects on the nurses’ level of professional stress” (30).

A significant difference was found in Predisposing, Reinforcing and Enabling Constructs in Educational Diagnosis and Evaluation Model (PERCED) constructs and stress management behaviors in the intervention group compared to the control group after a training intervention (P < 0.001) (23). The findings indicated a significant positive correlation between job stress and nurses; however, there was not a significant difference among nurses working in cardiology and pediatrics (24). There was a significant correlation between stress level, job satisfaction and leisure (29). The female nurses and the nurses with B.Sc. degree perceived (statistically significant) hazards of the workplace more stressful than male and M.Sc. nurses (25).

 There was a significant difference in the mean scores of coping strategies of stress focus, emotion focus, and ineffectiveness after yoga exercise compared with those before yoga sessions (28). There was a relationship between the use of the confronting coping scale and sex of the participants, and also a relationship was considered in the use of any coping scale and other demographic variables (22). A positive correlation was reported between the nurses’ ICU rating and gender (P = 0.013) and their degrees (P = 0.021) (25).

## 4. Conclusions

In this section, we conclude the findings briefly and relate the literature to the results of the 10 relevant articles. The earliest study was reported in 2007 (29), and the most recent study was reported in 2012 (30). This section

1. discusses the features of stress among nurses who are working in hospitals,
2. explores the potential positive or negative effects of stress and coping strategies on Iranian nurses,
3. describes instruments used to measure causes of stress, and
4. the effects of coping strategies and job satisfaction of Iranian nurses who were working in Iranian hospitals in different wards.

We identified only 10 studies, the majority of them (60%) analyzed the effect of coping strategies and experiences and perception of job-related stresses in Iranian nurses working in hospitals, the effect of a cognitive–behavioral stress management training program; the relationship of nurses’ stresses with environmental and occupational factors were rarely included at the time of our review. However, the reviewed studies were limited to the investigation of job stress and coping mechanisms among Iranian nurses in 28 hospitals.

A similar study was conducted by Gholamzadeh et al. (2011) who described occupational stress as a recognized problem in health-care workers. Nursing has been identified as an occupation that has high levels of stress (22). Burgess and Wallymahed (2010) found that certain personality trait such as openness and extraversion were associated with less perceived stress from the patient and relatives in nurses of Intensive Care Units (32).

This research showed that the majority of reviewed studies were used non-randomized sampling method. Moreover, the participants, who had enrolled in the reviewed studies, were working in different wards and had work experience. Furthermore, they were volunteer participants. However, the outcome measures were inaccurate. Perhaps using a random sampling method in selecting participants for stress management interventions should be considered in future researches to minimize bias due to volunteer participation. More authentic and convincing outcomes could therefore be found.

The review revealed several carefully developed instruments and analytic approaches for measuring stress, and the effects of coping strategies and job satisfaction on Iranian nurses in their working environment. While these measures were developed for a specific study, the nursing stress scale, which accounted for 30% of the stress measurements, was the most used strategy.

Three of the studies explored the relationship between job stress and job satisfaction. In this regard, the majority (74.4%) of nurses reported that they were satisfied. This instrument can provide a useful stepping stone for the further study of stress. In some studies, the measures were taken as part of an instrument; however, it is important to use them as measures to identify the degree of stress level, and to better understand their coping skills. Furthermore, it is also helpful to investigate the method of response to stress.

In contrast, Nabirye et al. (2012) found that there were significant negative relationships between occupational stress and job performance. Nurses in the public hospitals reported higher levels of occupational stress and lower levels of job satisfaction and performance (33). This finding has implications for the importance of improving the work environment to increase nurses’ job satisfaction (34).

The majority of this reviewed studies utilized both qualitative and quantitative approaches to answer the research questions. Meanwhile, a few studies were using only qualitative method which helps to collect, analyze, and interpret data. They used this method to assess phenomenon, process, prospective and views of Iranian nurses. Two studies, however, used quasi-experimental and interventional approaches. A possible reason for researchers’ preference for conducting non-experimental studies instead of interventional and experimental studies may be due to issues associated with ethics, and practicality exposing participants to stress, failure of their effects on coping strategies and job dissatisfaction.

A quality score based on nursing professional opinions with standard forms were used to obtain information according to the study design. The majority of reviewed studies frequently used descriptive statistics and analysis of variance (ANOVA) to compute study variables and socioeconomic characteristics. This is consistent with a study conducted by Sudhaker et al. (2010) who revealed moderate to high levels of stress in nurses. A better understanding of the demographic and work factors that lead to job stress should help managers understand better their employees’ satisfaction, performance and turnover, and consequently, help them to deal with stress (35).

Despite the variability of the measured outcomes, there were three main results associated with psychological factors measured by most of the studies: nursing stress scale (accounted for 30% of the studies), stress measurement (the most commonly used strategy anxiety), and depressive and psychological distress symptoms.

There was no significant correlation between stress level and some demographic information; also there was no significant correlation between quality of life or occupational stress and factors like position, shift, ward, experiences, time off, over time hours, interest in desertion and education.

These outcomes were mainly measured by established psychological health measures such as the Nursing Stress Scale; Strauss and Corbin’s grounded theory; Coping stress Revised questionnaire; Lazarus standard questionnaire; author-developed questionnaire based on RECEDE Model; Persian short Version genetic Job stress National Institute for Occupational Safety and Health (NIOSH); Job stress, residency, Enzyme -linked immunosorbent assay (ELISA); Burnout Questionnaire and Toft Gray and Anderson’s tool; open semi-structured Interview; and general demographic characteristics for measuring some related causes of stress in Iranian nurses in their working environment.

This finding is consistent with by Currid (2008) who found that in implementing strategies to improve acute wards, it is important to look at the well-being of staff (those who care the patients in this specific area of nursing) (36). Regarding the stress, effect of coping strategies, and job satisfaction, these reviewed studies revealed that the coping strategies of Iranian nurses had important positive outcomes in several health areas. The reported positive outcomes were associated with the positive Iranian nurses’ response; reduced degree of stress; improved job satisfaction; decline in mood disturbances; improved coping skills; increased awareness about stress, its effects and management; and improved perceived ability to cope effectively and positively.

Although, improvement of perceived ability (like yoga and other stress coping strategies) to cope effectively has positive effects among Iranian nurses; it should be interpreted with precaution. As the number of studies was small and there might be a methodological impact too. Likewise, in the study conducted by Wang et al. (2009), they concluded that in order to reduce and manage nurses’ job stress, first we need to recognize the impacts of job-related stress and effective coping methods (37).

The implications of this review are significant in certain areas which worth further research. Future research must look at the impacts of different types of stress with respect to duration and frequency, effect of coping strategies, the impact of job satisfaction on Iranian nurses’ health, and personal and professional development. Therefore, the optimal duration and frequency of these coping strategies to create positive impacts can be determined.

It is also worthy to explore which components of these coping strategies create job satisfaction effects and which are more targeted. In addition, future research must find the best coping strategy for each group of nurses in different health settings. Consequently, personalized and customized stress management, effects of coping strategies and job satisfaction in the work environment can be designed accurately and effectively.

The effect of yoga as a coping strategy on Iranian nurses’ stress was regarded a positive outcome, but it needs to be interpreted with caution. Further research to address long-term effects of yoga and the underlying systemic mechanism leading to its stress reduction mechanism, improvement of coping strategies and skills should be conducted.

Finally, future researches must use rigorous methodology to elicit the real impacts of stress, effects of coping strategies and job satisfaction among Iranian nurses. Suggestions for future research are also mentioned. Perhaps the implications discussed in this review are not only confined to medical students, but also can be utilized by researchers of other disciplines as a guideline to design, plan and conduct similar researches in their own settings. Use of similar health measurements for comparison of outcomes in the future researches is recommended.
